# Quantum anomaly detection for collider physics

**DOI:** 10.1007/JHEP02(2023)220

**Published:** 2023-02-22

**Authors:** Sulaiman Alvi, Christian W. Bauer, Benjamin Nachman

**Affiliations:** 1 Department of Physics, University of California, Berkeley, CA 94720 USA; 2 Physics Division, Lawrence Berkeley National Laboratory, Berkeley, CA 94720 USA; 3 Berkeley Institute for Data Science, University of California, Berkeley, CA 94720 USA

**Keywords:** Multi-Higgs Models, New Light Particles

## Abstract

We explore the use of Quantum Machine Learning (QML) for anomaly detection at the Large Hadron Collider (LHC). In particular, we explore a semi-supervised approach in the four-lepton final state where simulations are reliable enough for a direct background prediction. This is a representative task where classification needs to be performed using small training datasets — a regime that has been suggested for a quantum advantage. We find that Classical Machine Learning (CML) benchmarks outperform standard QML algorithms and are able to automatically identify the presence of anomalous events injected into otherwise background-only datasets.

ArXiv ePrint: 2206.08391

## Contents

**1 Introduction** **1**

**2 Quantum Machine Learning** **2**

**3 Simulation** **3**

**4 (Quantum) Machine Learning Setup** **5**
- 4.1 VQC 6
- 4.2 QCL 6
- 4.3 CML 7

**5 Results** **7**

**6 Conclusions** **10**

**A Max(SIC) plots** **12**

JHEP02(2023)220

## 1 Introduction

Quantum computing is a promising tool for a variety of problems because an exponentially large Hilbert space can be described by polynomially many qubits. In high energy physics, there is particular promise for simulations of quantum field theories, where every spacetime point has quantum degrees of freedom, but polynomial algorithms exist for state preparation and time evolution [1, 2]. However, not all classically hard algorithms are more efficient on a quantum computer.

One particular class of algorithms that has received significant attention in high energy physics (HEP) is Quantum Machine Learning (QML). In this paper, QML refers to machine learning tasks that are executed on quantum computing hardware. While QML is not known to be more efficient than classical machine learning (CML), there have been many empirical studies to explore the potential of QML for HEP [3–19] (see also ref. [20] for a recent review).

A common theme that has emerged from these studies is that QML seems to outperform CML with small training datasets.[1] While there is no rigorous explanation for this observation, it could be that QML provides a superior inductive bias and/or more expresivity with a smaller number of parameters. In nearly all studies, CML outperforms QML when there are more than $\mathcal{O}(100)$ examples. There are almost no problems in collider HEP that have such small numbers of events for training. The goal of this paper is to explore a realistic use case for near-term QML for collider physics. See also ref. [21] for the broader context of QML versus CML.

[1]There may be other advantages as well, such as the prospect for more global optimization instead of local optimization. We leave this exploration for future studies.

Most analyses at the Large Hadron Collider (LHC) make use of simulation for training classifiers. Since these simulations can be used to generate more events independent of collider operations, they are not in a regime where QML is expected to outperform CML with near-term quantum hardware. Therefore, analyses that have the potential for QML to outperform CML should require data for training. Since we do not know the origin of any particular data event, such methods are called *less than supervised* [22].

One particularly promising class of less-than-supervised methods is signal model-independent anomaly detection. Such approaches are characterized by the comparison of data in a particular region of phase space (*signal region*) with a reference sample. There have been many proposals for doing this comparison using machine learning (for overviews, see e.g. refs. [22–24]). A highly sensitive approach is to train a classifier to distinguish data from a precise prediction of the background (semi-supervised learning). If the background is well-understood theoretically, then the reference sample could be simulation. This has the advantage that the background prediction does not need to be learned, but has the disadvantage of being strongly background-model dependent.

There are few final states at the LHC for which the background is known precisely enough to be used directly for background estimation. One exception is the final state with four charged leptons. Both ATLAS [25–27] and CMS [28–30] directly use Monte Carlo (MC) simulations to estimate the background and ATLAS even uses machine learning to isolate particular signals [25]. While powerful, this approach is signal model-specific and does not readily extend to models with multidimensional parameters. Reference [31] recently proposed to use machine learning as an alternative approach. It was shown that training classifiers to distinguish data from background-only simulation provides a complementary approach to direct searches and has broad signal sensitivity. As the four lepton final state also has a small cross section, this is a natural target for studying QML.

The prospect of QML for anomaly detection was first studied in ref. [16] in the context of autoencoders. These unsupervised tools can be trained without any simulation, but are not as effective as semi-supervised methods when there is a good background model and/or when the new physics is not the lowest density events [32, 33]. For this reason, our focus is on semi-supervised learning. We also assume an idealized situation where there are no systematic uncertainties. Nuisance parameters will likely not change the qualitative conclusions of the QML versus CML comparison and have been discussed in ref. [34] for anomaly detection.

This paper is organized as follows. Section 2 briefly introduces various QML approaches. The simulated samples to be used for the machine learning are described in section 3. Numerical results are presented in section 5 and the paper ends with conclusions and outlook in section 6.

## 2 Quantum Machine Learning

There are a number of ways quantum computers can be used for machine learning. One of the first applications of quantum machine learning to a high energy physics problem explored quantum annealing for identifying Higgs-boson events [3]. Another possibility is that quantum algorithms can reduce the computational complexity of linear algebra

JHEP02(2023)220

operations core to a number of ML approaches (see e.g. refs. [35–37]). The approach we focus on involves using quantum circuits as flexible function approximators similar to neural networks and other classical ML techniques. Quantum-classical variational methods used to optimize parameteric circuits are the analog of tuning the weights and biases of a classical neural network. These circuits have been applied identifying SUSY events [6], $t\bar{t}H$ and $H \to \mu^+\mu^-$ analyses [8], and $Z'$ resonance searches [5].

There have been a number of claims in the literature that QML methods of this kind outperform CML for limited training data. While there is no formal proof of this claim, it could be justified intuitively as a result of the improved *inductive bias* of quantum circuits. In other words, the class of functions that QML represent with a limited set of parameters are more relevant / tailored to HEP problems. We aim to explore this claim in the context of two variational algorithms in the four lepton anomaly detection search.[2]

The two QML methods we study are called Variational Quantum Circuits (VQC) and Quantum Circuit Learning (QCL). These are both implementations of parameterized quantum circuits where the various rotation angles are optimized via classical methods. Each algorithm is composed of multiple components: state preparation, which encode classical data into quantum states, the model circuit, which contains the parameters that are optimized during the training process, and the measurement and output, which are used to evaluate performance of the circuit. VQC and QCL differ only in the structure of the parameterized circuit, as detailed in section 4. As the examples we study in this paper are relatively small in terms of quantum resources, all circuits are simulated on classical computers.

Note that we also studied Quantum Support Vector Machines (QSVMs) [36], but initial tests suggested that they are strictly less effective than other methods so they were not included in the final tests. See also ref. [38] for a broader perspective on QVSMs. We have also explored the quantum gradient descent studied in ref. [5]. We tested the setup using `Pennylane` [39] and found that the learning rates were unreliable for Gaussian classification as well as our HEP application, so this was not pursued further.

## 3 Simulation

The simulated datasets are the same as in ref. [31] and are briefly summarized in the following. All events are generated with MadGraph5_aMC@NLO 2.8.0 [40]. Both signal and background events are generated using the Higgs Boson Effective Field Theory (`heft`) [41] in which the heavy top quark limit is used for the gluon-gluon-Higgs vertex. We focus on the $e^+e^-\mu^+\mu^-$ final state to avoid combinatoric ambiguity (see figure 1). After the matrix element calculations, the outgoing particles are processed with Pythia 8.244 [42–44] with its default settings for parton showering and hadronization. Pythia also handles the decay of the anomalies, which are Higgs-like scalar particles with a mass of 125 GeV decaying asymmetrically into two lighter-mass bosons, one of which decays to electrons and the other which decays into muons. This is accomplished technically by generating Higgs

[2] As a cross-check, we also explored the Supersymmetry example of ref. [6]. We were unable to reproduce the QML superior performance at low sample size and the CML was also significantly improved by adding more parameters.

JHEP02(2023)220

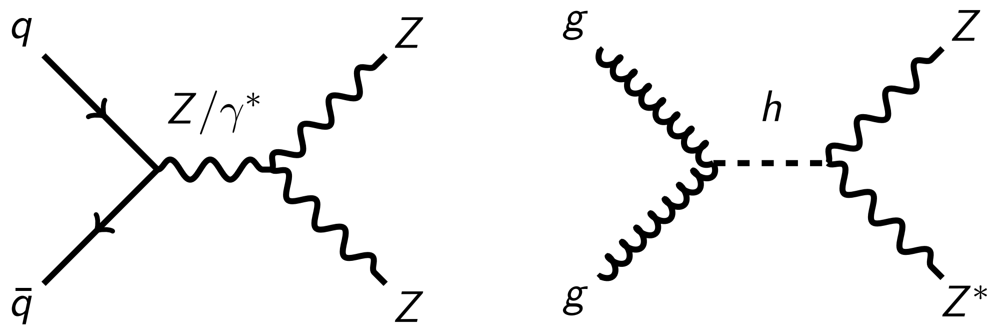

**Figure 1**. Left: an example background leading order Feynman diagram and Right: an example signal Feynman diagram. One of the outgoing $Z$ bosons decays to muons and the other decays to electrons.

JHEP02(2023)220

bosons and then replacing the PDGID [45] of 25 (SM Higgs) with 35 (2HDM heavier Higgs) in the Les Houches Event (LHE) files [46] and then setting the decay of this particle into particles with PDGIDs 23 (Z boson) and 36 (2HDM pseudoscalar). Subsequently, the $Z$ boson (pseudoscalar) is forced to decay into electrons (muons). All three BSM particles are set to a narrow width. The detector response is emulated with Delphes 3.4.2 [47–49] using the default CMS card. We will make the simplifying assumption that there is no systematic uncertainty in the background estimation and so the 'data' and 'simulation' are statistically identical when no signal events are injected. Recent studies exploring the integration of systematic uncertainties in this setup can be found in ref. [34]. The number of events in background corresponds to the LHC Run 2 dataset (about $150\,\text{fb}^{-1}$, which corresponds to thousands of events that pass our event selection).

In this work we focus only on the leptoninc final states. Other event properties could also be useful, however, information about the hadronic final state is known with less precision and thus may introduce the need to go beyond a pure simulation-based background estimation. Each event is characterized four three-momenta (12 numbers in total). For our target models, $pp \to A \to B(\to e^+e^-)C(\to \mu^+\mu^-)$, the three masses $m_{e^+e^-\mu^+\mu^-}, m_{e^+e^-}, m_{\mu^+\mu^-}$ are nearly sufficient[3] statistics for characterizing the new physics. In this paper, we consider signals of this form, where $A$ is a non-SM Higgs boson that decays to two different mass bosons. The model is specified by three parameters: $m_A, m_B$, and $m_C$. There is also an overall cross section set by the coupling of the $A$ particle to the rest of the Standard Model. This cross section will be varied in the subsequent analysis by considering different numbers of signal events. Given the three-dimensional parameter space, we focus on the three-dimensional problem in this paper. Non-resonant signals and signals with non-trivial spin structures could benefit from using more of the phase space in the future. While there are currently LHC searches for the case that $B = C$ (or $B$ is a BSM particle and $C$ is a $Z$ boson), there is currently no search where all three masses could be different (although not for a lack of physics motivation [50]).

The spectra of the three invariant masses ($m_{e^+e^-\mu^+\mu^-}, m_{e^+e^-}, m_{\mu^+\mu^-}$) for the background and our representative signal are presented in figure 2. As expected, the di-electron

[3]In Statistics, a sufficient statistic is an observable that contains all of the relevant information for inference.

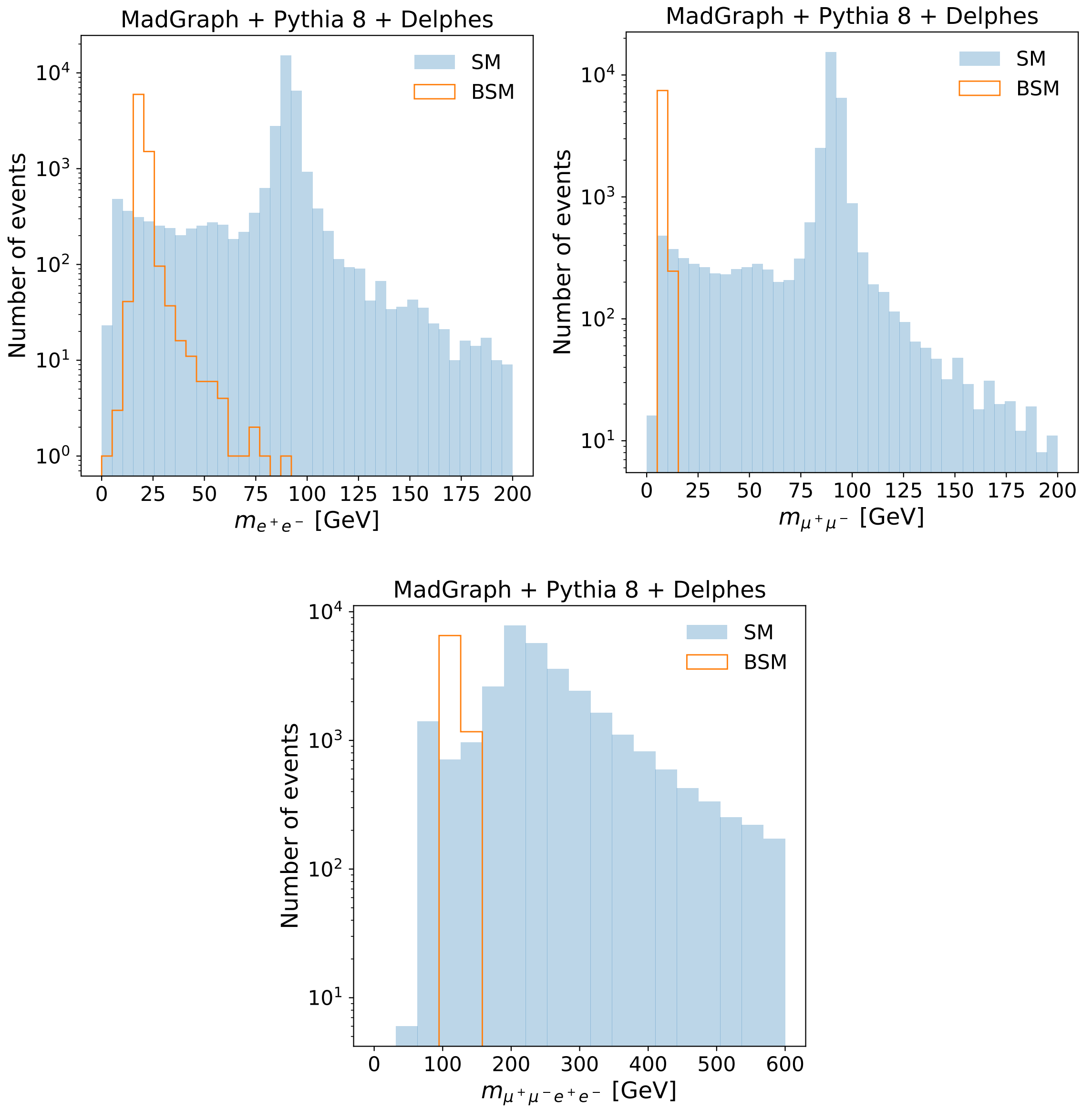

**Figure 2**. The three dimensions used for machine learning: $m_{e^+e^-}$ (left), $m_{\mu^+\mu^-}$ (right) and $m_{4\ell}$ (bottom).

and di-muon invariant masses peak near the $Z$ boson mass of 90 GeV [45] and there are peaks in the four-lepton invariant mass at the $Z$ peak and the Higgs boson mass of about 125 GeV [45]. The signal is resonant in all three observables with peaks at the masses of the particles. The parent particle (125 GeV mass) decays to two children, with masses of 25 and 15 GeV for electrons and muons, respectively. Note that these parameters are not known to the neural network.

## 4 (Quantum) Machine Learning Setup

As stated earlier, our QML approaches are both variational circuits that are analogous to classical neural networks with a large number of tunable parameters. We consider two

JHEP02(2023)220

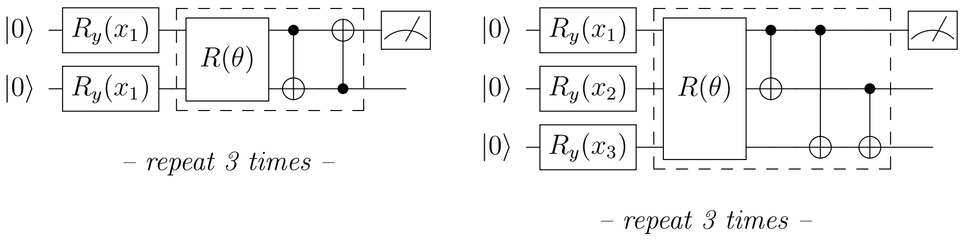

**Figure 3**. VQC circuit for the one-dimensional (left) and three-dimensional (right) datasets.

flavors: VQC and QCL. These two approaches are described in more detail below and only differ in how the classical data are encoded and what parameterized circuits are used in the learning. VQC uses a simpler encoding with a multi-qubit rotation followed by a series of CNOT gates for the variational part. Instead, QCL uses a more complex encoding and then time evolution of a certain Hamiltonian for the variational component.

### 4.1 VQC

Figure 3 shows the VQC circuits used in this study. The input features ($x_i$) are min-max scaled so that the argument of the initial $R_y$ gates are valid angles. The rotational gate $R(\theta)$ is given by $R_Z(\alpha)R_Y(\omega)R_Z(\phi)$, where $\alpha$, $\omega$, and $\phi$ are the trainable weights of the circuit. These angles are unique for each qubit, leading to a total of 18 parameters for the one-dimensional setup and 27 for the three-dimensional setup. The output is the expectation value of $Z$, which is achieved by taking several shots of each qubits.

There are a number of hyperparameters that must be selected and the ones described above were chosen after a detailed study. By varying the number of qubits, we observed that two qubits resulted in an optimal accuracy for the one-dimensional classification task. Using more than six qubits caused issues related to overfitting of the data while also increasing runtime. As such we chose three qubits for the three-dimensional case. We observed that we needed a minimum of three rotation gates in order to achieve an effective level of expressivity of the circuit. Between three and five rotation gates, we did not see a significant increase in accuracy, and beyond five rotation gates, the time to run the simulation became prohibitive while also not resulting in an increase in performance. The default encoding gate included a $Y$ and $Z$ rotation but testing different combinations of input encoding gates showed no performance difference so the final circuit just included a $Y$ rotation gate. Batch learning was also implemented for the three-dimensional dataset tests, which greatly improved performance. The circuit was optimized using vanilla gradient descent, which is equivalent to classical stochastic gradient descent with the gradient of the quantum circuit calculated using `Pennylane`'s [39] implementation of the parameter shift rules for quantum circuits.

### 4.2 QCL

The circuit's parameterized gates follow [6] in a way that allows for access of the entire Bloch sphere given an arbitrary input state. The gate that introduces entanglement involves a time evolution operation following the Ising model Hamiltonian $H$ as in figure 4. We did

JHEP02(2023)220

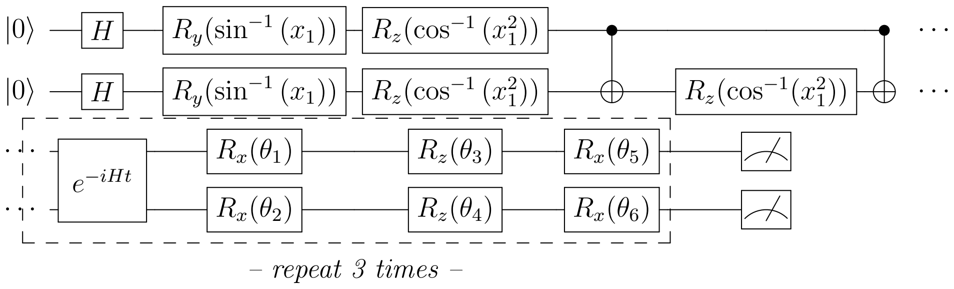

**Figure 4**. QCL circuit for 1 dimensional datasets.

not experiment with different Hamiltonians as their work remarks that changing this did not achieve a different result. The expectation value $z_i$ of the Pauli Z operator for the first two qubits is measured and the loss function is cross-entropy:

$$-\sum\left[\log\left(\frac{e^{z_0}}{e^{z_0}+e^{z_1}}\right)+\log\left(\frac{e^{z_1}}{e^{z_0}+e^{z_1}}\right)\right], \tag{4.1}$$

where the exponential terms represent the soft-max activation function. The number of qubits to encode parameters in the 1D case was chosen after a brief exploration to be two. Each input value was duplicated and stored in two separate qubits which were transformed by the same circuit. For 3D testing we used six qubits. For all tests the circuits had three layers, which are outlined in figure 4. The optimization was completed using the `COBYLA` method [51] with the parameter shift rule as defined in [6] and binary cross-entropy loss.

The $\theta_i$ are the trainable weights of the circuit. The three-dimensional case follows the same structure as figure 2 and the alternative $U_{\mathbf{in}}$ in figure 10 from [6], except the three inputs are duplicated so that six qubits can be used. There were 18 trainable parameters for the one-dimensional setup and 54 trainable parameters for the three-dimensional setup.

### 4.3 CML

The performance of the quantum machine learning setups are compared against two neural networks implemented in `TensorFlow` [52]. The first network ('NN Low') has one layer with 32 nodes and the second network ('NN High') had two 32 node layers, which corresponds to 97 and 1,217 trainable parameters, respectively. The output was passed through a sigmoid activation and the NN High model used rectified linear activation (ReLU) functions for the intermediate layer.

The networks were optimized using the binary cross entropy with `Adam` [53]. Each network was run for a fixed number of epochs, determined by the convergence of the loss curves: 50 epochs for the one-dimensional example and 100 for three-dimensional example. A summary of the various ML models is presented in table 1.

## 5 Results

We perform a weakly/semi-supervised search where a classifier is trained to distinguish a background sample from another, statistically independent and identical sample, that

JHEP02(2023)220

| 1D | Model | Parameters | Time (h:mm:ss) | Optimization Method | Optimization Steps |
|---|---|---|---|---|---|
| | VQC | 18 | 0:56:42 | Vanilla GD | 50 |
| | QCL | 18 | 0:00:45 | COBYLA | 30 |
| | NN Low | 97 | 0:03:25 | Adam | 50 |
| | NN High | 1217 | 0:03:40 | Adam | 50 |
| 3D | | | | | |
| | VQC | 27 | 1:57:35 | Vanilla GD | 50 |
| | QCL | 54 | 0:01:07 | COBYLA | 20 |
| | NN Low | 97 | 0:06:40 | Adam | 50 |
| | NN High | 1217 | 0:03:35 | Adam | 100 |

**Table 1**. A summary of the hyperparameters chosen for each classifier and average running (training and testing) time. The times were evaluated using the Perlmutter computer at NERSC, which has nodes consisting of A100 NVIDIA GPUs (used for NNs) and Milan AMD CPUs.

has some number of signal events added to it. Due to the small number of injected signal events, it is important to study the sensitivity to different random sets of signal events. The results presented below are averaged over 80 different random selections of signal and background (with error bars representing the standard deviation). The performance is quantified in terms of the number of standard deviations[4] achieved after applying the optimal (maximum significance improvement) threshold on the network.[5] The maximum significance improvement is model dependent, but it is chosen here to bound the achievable performance. In practice, the challenge of selecting the threshold is the same for both QML and CML methods. Two relevant benchmark significance are $2\sigma$ and $5\sigma$ which approximately correspond to the community standards for excluding and discovering a model, respectively.

Numerical results are presented in figure 5. As a first test, we fix the background at 1000 events and scan the number of signal events. For reference, the naive significance with 30 signal events is about unity. All of the methods are better than doing nothing and surpass $2\sigma$ by 40 events. The QML models do not outperform the classical approaches and in fact appear to be systematically worse except perhaps for the lowest number of signal events where the significances themselves are below unity (and thus irrelevant). Similar trends hold for the three-dimensional data, except that the QCL performs relatively worse and the NN High performs relatively better. We note that the error bars on the plots are not small, which illustrates the importance of ensembling over the random parts of the training as well as over the random injetion of signal (and background) events.

As a second test, we fix the signal fraction and vary the number of signal events (which also changes the number of background events). At 1% fraction, for reference, 10 signal events would then correspond to the 1000 background events in the lefthand plots of figure 5.

[4]This is approximated by the number of signal events divided by the square root of the number of background events. We do not expect that more precise calculations/approximations will qualitatively change the results.

[5]Other papers have used the Area Under the Receiver Operator Characteristic Curve (ROC AUC). While the AUC is a standard metric in machine learning, it is not representative for HEP applications where typically one working point (threshold) on the classifier is used instead of integrating across the entire ROC curve.

JHEP02(2023)220

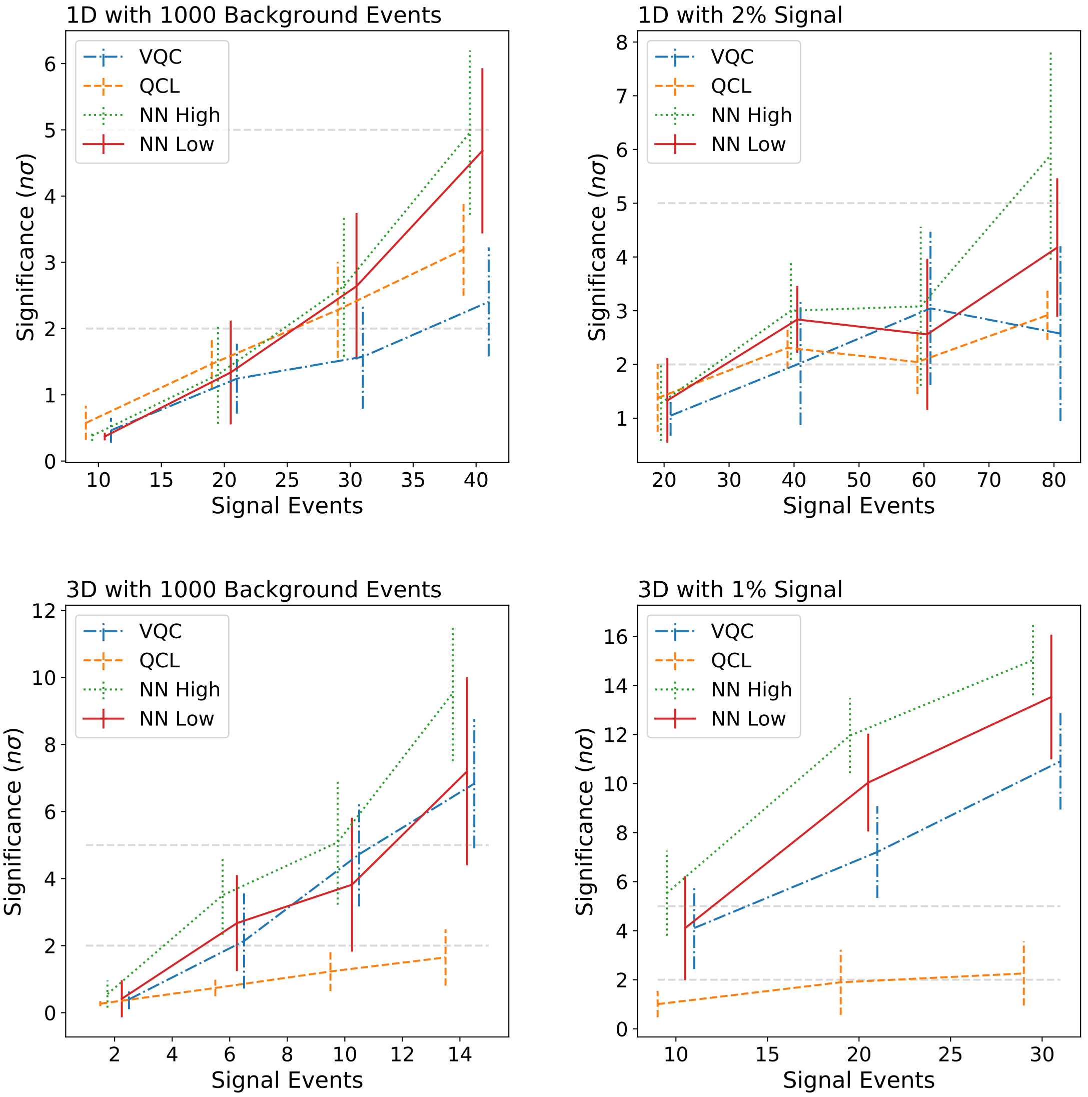

**Figure 5**. Results plotted against increasing signal events. The horizontal grey lines indicate thresholds for exclusion ($2\sigma$) and discovery ($5\sigma$). The starting significance (i.e. significance before making any cuts) for each graph is: Top Left: { 0.3, 0.6, 0.9, 1.3 } Top Right: { 0.6, 0.9, 1.1, 1.3 } Bottom Left: { 0.06, 0.2, 0.3, 0.4 } Bottom Right: { 0.3, 0.4, 0.5 }.

The trends for the fixed signal fraction are similar to the fixed background fraction, with slightly worse performance due to the larger number of background events beyond 10 signal events. Additional performance metrics including the Area Under the ROC curve (figure 6) and the relative significance improvement can be found in appendix A. These additional statistics corroborate the story presented in figure 5. The AUC is difficult to interpret as it integrates across the entire ROC curve when in practice, we typically operate at a fixed working point. Small changes in the AUC could correspond to regions of the ROC curve that are physically useful, so the maximum significance improvement is instead chosen as the default statistic.

JHEP02(2023)220

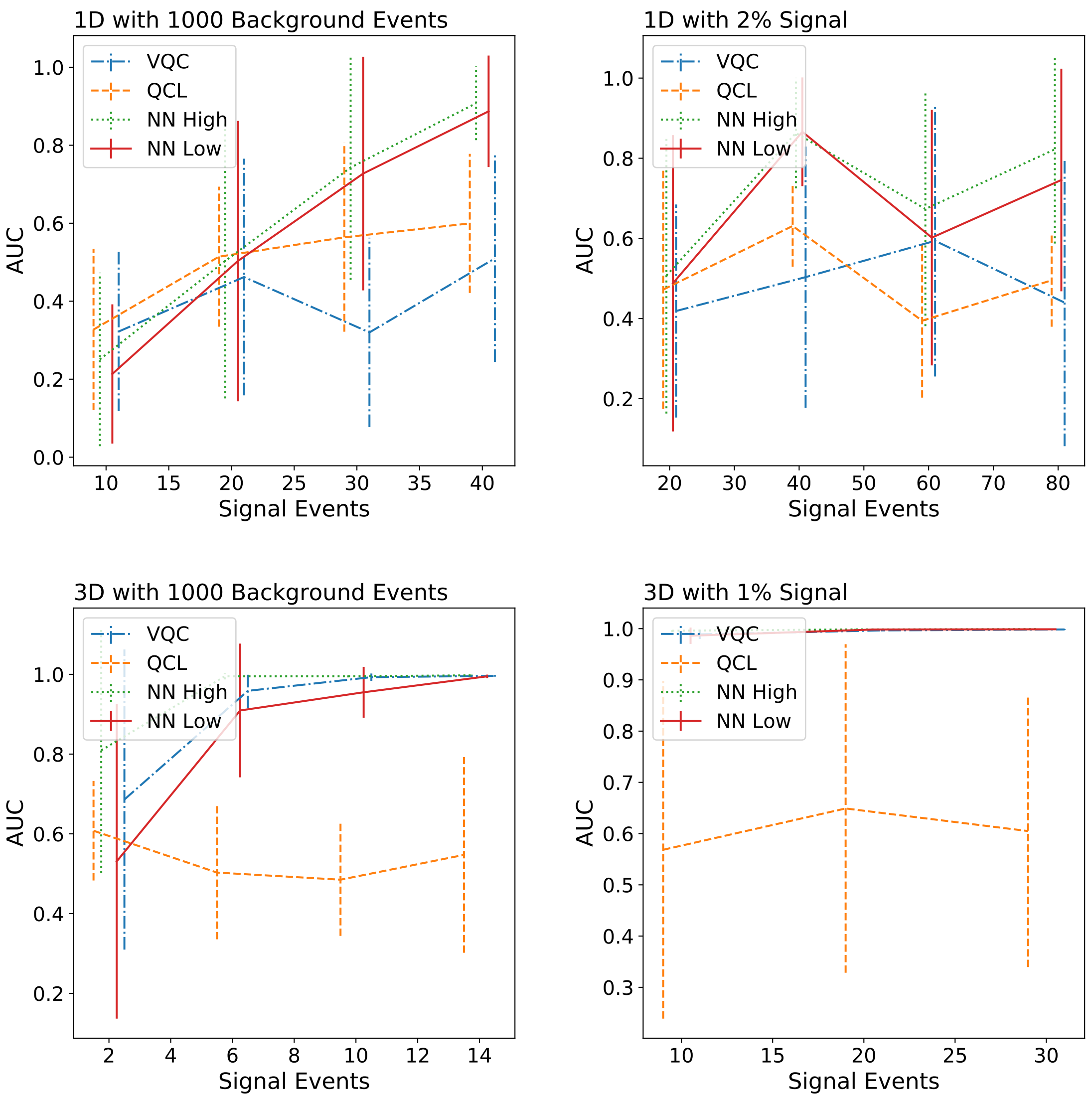

**Figure 6**. AUC plotted against increasing signal events.

## 6 Conclusions

Motivated by promising numerical studies from the HEP literature on quantum machine learning for low-event count applications, we have explored where these techniques could be practically useful for collider physics. Given that most machine learning methods are trained using simulation (which are not limited in statistics), we concluded that a task relying directly on data should be the target application. An important topic in this area is anomaly detection, where machine learning methods are used to reduce model dependence. One strategy that reduces signal model dependence is to train a classifier to distinguish data from background-only simulation. This will be fundamentally limited by the number of events in data and thus may be a good target for exploring an advantage of quantum machine learning over its classical counterpart.

JHEP02(2023)220

There are not many final states that are modeled precisely enough for the data versus simulation strategy to be effective at finding small signals. Following ref. [31], we consider anomaly detection in the four-lepton final state, where the Standard Model is well-known, yet there is plenty of phase space for new physics. We consider a low-dimensional version of the problem within a model framework that has three free parameters (masses), which already extends beyond the current searches at the LHC that focus on one and sometimes two-dimensional versions of the problem. We do not find any advantage of quantum machine learning over classical machine learning.

It could be that this particular problem is not well-suited for quantum machine learning or that we have not picked exactly the right quantum machine learning architecture or training process. We do not claim that our results are general for all of QML and all of HEP, but we hope that our process and numerical results will be useful to put existing and future studies of QML for HEP in context.

## Acknowledgments

This work was supported by the Department of Energy, Office of Science under contract number DE-AC02-05CH11231. S.A. was also supported by a University of California Summer Undergraduate Research Fellowship. This research used resources of the Oak Ridge Leadership Computing Facility, which is a DOE Office of Science User Facility supported under Contract DE-AC05-00OR22725. We thank Vinicius Mikuni for computing help and Andrew Blance, Koji Terashi, and Michael Spannowsky for useful discussions about QML.

JHEP02(2023)220

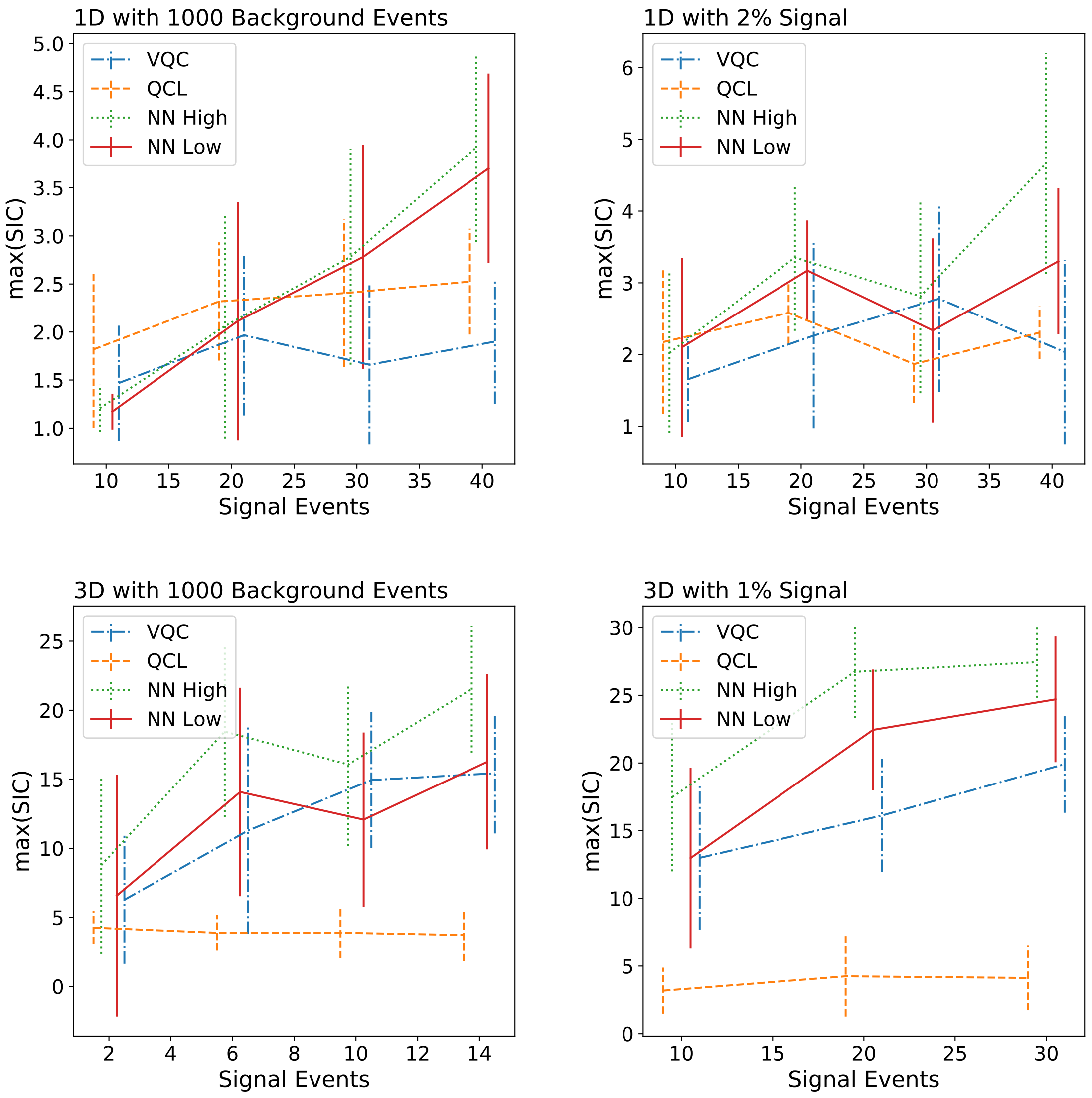

**Figure 7**. The maximum significance improvement as a function of the number of signal events.

## A Max(SIC) plots

Figure 7 quantifies the performance of the same studies as in section 4, using maximum significance improvement.

**Open Access.** This article is distributed under the terms of the Creative Commons Attribution License (CC-BY 4.0), which permits any use, distribution and reproduction in any medium, provided the original author(s) and source are credited. SCOAP$^3$ supports the goals of the International Year of Basic Sciences for Sustainable Development.

JHEP02(2023)220
